# Parenteral nutrition during neoadjuvant chemotherapy for patients with non-metastatic gastric or esophago-gastric cancer to reduce postoperative morbidity (PERCOG): study protocol for a randomized controlled trial

**DOI:** 10.1186/s13063-017-2388-3

**Published:** 2017-12-28

**Authors:** Tara C. Mueller, Rebekka Schirren, Victoria Kehl, Helmut Friess, Daniel Reim, Marc E. Martignoni

**Affiliations:** 1 Department of Surgery, Technical University of Munich School of Medicine Klinikum rechts der Isar, Ismaninger Straße 22, 81675 München, Germany; 2 Institute for Medical Statistics and Epidemiology, Klinikum rechts der Isar, Technische Universität München, Ismaninger Straße 22, 81675 Munich, Germany

**Keywords:** Gastric cancer, Cancer of the esophago-gastric junction, Neoadjuvant chemotherapy, Parenteral nutrition, Postoperative complications, Comprehensive Complication Index, Randomized controlled trial

## Abstract

**Background:**

The majority of patients with gastric or esophago-gastric cancer are at risk for malnutrition. Preoperative malnutrition was shown to increase the incidence of postoperative complications following abdominal surgery. However, it remains unclear if preoperative parenteral nutritional support during neoadjuvant chemotherapy (NACT) may be effective to reduce the rate of postoperative complications in these patients.

**Methods/Design:**

The PERCOG trial is a randomized controlled multicenter observer-blinded trial, investigating if the improvement of the general condition of patients with non-metastasized gastric cancer or cancer of the esophago-gastric junction during NACT by supplemental parenteral nutrition can decrease the postoperative Comprehensive Complication Index (CCI). Statistical analysis of the primary endpoint measure (CCI on postoperative day 30) will be based on the intention-to-treat population. The global level of significance is set at 5% and the sample size (n = 150) is determined to assure a power of 80%.

**Discussion:**

The results of the PERCOG trial will provide high-level evidence for clinical recommendations regarding the administration of preoperative supportive parenteral nutrition and provide all participating patients the opportunity of an improved treatment.

**Trial registration:**

German Clinical Trials Register, DRKS00009451. Registered on 3 July 2017.

**Electronic supplementary material:**

The online version of this article (doi:10.1186/s13063-017-2388-3) contains supplementary material, which is available to authorized users.

## Background

### Rationale

Malnutrition affects the majority of surgical oncological patients and negatively influences the postoperative outcome. In particular, patients with cancer of the upper gastrointestinal tract (GIT) have an increased risk for malnutrition-associated, postoperative complications. Malnutrition weakens the immune system, which in turn increases the risk for infections and impaired wound healing. In addition, postoperative hypermetabolism leads to an increased energy and protein demand, which can result in or contribute to malnutrition of these vulnerable patients [1]. In patients with adenocarcinoma of the stomach or the esophago-gastric junction staged cT2 cN+ cM0 up to cT4 cN+ cM0, neoadjuvant chemotherapy (NACT) followed by the oncological resection of the tumor is the standard therapy concept in Germany [2]. The PERCOG trial aims to resolve the question if supportive parenteral nutrition during the NACT of these patients can decrease the incidence of postoperative complications following the oncological tumor resection. Reporting of postoperative complications in surgical trials is often non-standardized and thus results are difficult to assess and compare between trials. Therefore, the chosen primary endpoint of the PECOG trial is the Comprehensive Complication Index (CCI), which includes all types of postoperative complications independent of their severity and has been shown to be more sensitive than other existing morbidity endpoint [3–5].

### Previous trials

Several studies previously investigated the effect of preoperative nutritional interventions on the postoperative outcome after abdominal surgery. However, their results are not unequivocal. A multicenter cohort study of 512 patients showed that preoperative supportive nutrition significantly reduced the risk of postoperative complications after abdominal surgery in patients at risk for malnutrition (Nutritional Risk Score [NRS 2002] ≥ 5) [6]. In these patients, the preoperative nutrition intervention also significantly reduced the length of postoperative hospital stay [7]. Many other studies did include only small patient numbers and/or showed methodological flaws and/or did not investigate clinically relevant endpoints. In a meta-analysis of 13 randomized controlled trials (RCTs), preoperative parenteral nutrition reduced the rate of postoperative complications about 10% [8]. However, the included trials differed substantially in included indications, operations, and endpoints. In another meta-analysis of 26 RCTs, it was shown that preoperative lipid-free parenteral nutrition reduced the rate of postoperative complications in malnourished patients [9]. However, the definitions of malnutrition in the included studies varied largely. A systematic review and meta-analysis of the Cochrane Group also showed that preoperative parenteral nutrition of malnourished patients reduced the risk for severe postoperative complications [10]. Significantly, only three RCTs were included in this meta-analysis, since all other trials failed to fulfil the required quality of reporting. Finally, another systematic review of 41 trials (including non-RCTs) did not support these results. Even if a slightly reduced rate of postoperative complications was observed in the group of patients receiving preoperative parenteral nutrition, it failed to achieve statistical significance [11]. In addition, several studies investigated the question, which surgical patients could especially benefit from preoperative nutritional support. Results from two older trials suggest that patients with malignancies of the upper GIT could especially benefit from preoperative nutritional support [12, 13]. These results were confirmed by a more recent RCT, in which the subgroup of patients with malignancies of the GIT showed the most significant effect of the preoperative nutritional support [14].

In conclusion, the existing evidence implies that preoperative parenteral nutrition can reduce the rate of postoperative complications after surgery for malignancies of the upper GIT. This group of patients is exposed to an increased risk of malnutrition because of the reduced food intake due to the anatomical location of the tumor, as well as nausea and loss of appetite, which are frequently caused by the NACT. Therefore, many of these patients experience clinically relevant loss of weight during NACT.

### Objective

This multicenter clinical RCT aims to improve the general condition of patients with non-metastasized gastric cancer or cancer of the esophago-gastric junction during NACT by supportive parenteral nutrition, in order to decrease the postoperative CCI [5]. The results of the PERCOG trial will provide high-level evidence for clinical recommendations regarding the use of preoperative supplemental parenteral nutrition and provide all participating patients the opportunity of an improved treatment.

## Methods/Design

### Trial sites

The PERCOG trial will be conducted in at least four surgical departments (university hospitals), all of which are members of the German surgical trial network (CHIR-*Net*) and have previously participated in multicenter trials within this network. All of the study personnel involved in the trial require training according to the International Conference on Harmonisation of Technical Requirements for Registration of Pharmaceuticals for Human Use – Good Clinical Practice (ICH-GCP) and will be specifically instructed in all trial-specific procedures before initiation of the trial, conforming to German Drug Law (*Arzneimittelgesetz*– AMG [15]) and GCP regulations [16]. After training by the principal investigator, the local home care service will administer the parenteral nutrition to patients according to their standard procedures.

### Trial population and eligibility criteria

All adult patients (aged > 18 years) with histologically confirmed adenocarcinoma of the stomach or the esophago-gastric junction staged cT2 cN+ cM0 up to cT4 cN+ cM0 will be eligible if they are able to understand the extent and nature of the PERCOG trial and provide written informed consent to participate.

Exclusion criteria were defined as: (1) non-resectable primary tumor, judged by the treating surgeon; (2) pregnant or breast-feeding women; (3) Eastern Cooperative Oncology Group (ECOG) score > 2; (4) NRS < 3; (5) insufficient liver, kidney, or bone marrow function, judged by the treating oncologist; (6) manifest cardiac illness (unstable coronary artery disease, heart failure New York Heart Association [NYHA] class IV) or respiratory illness (chronic obstructive pulmonary disease [COPD] class IV) that represent a contraindication either for the NACT or the surgical resection of the tumor; (7) participation in another interventional clinical trial that interferes with the primary or secondary outcomes of this trial; and (8) insufficient patient compliance.

### Sample size

A total of 150 patients have to be recruited (for calculation, see “Statistical methods”), 75 patients per group.

### Type of trial

This is an observer-blinded, multicenter, superiority surgical RCT with two parallel study groups, phase III-b according to German Drug Law (*AMG*).

### Recruitment and trial timeline

**Fig. 1:**
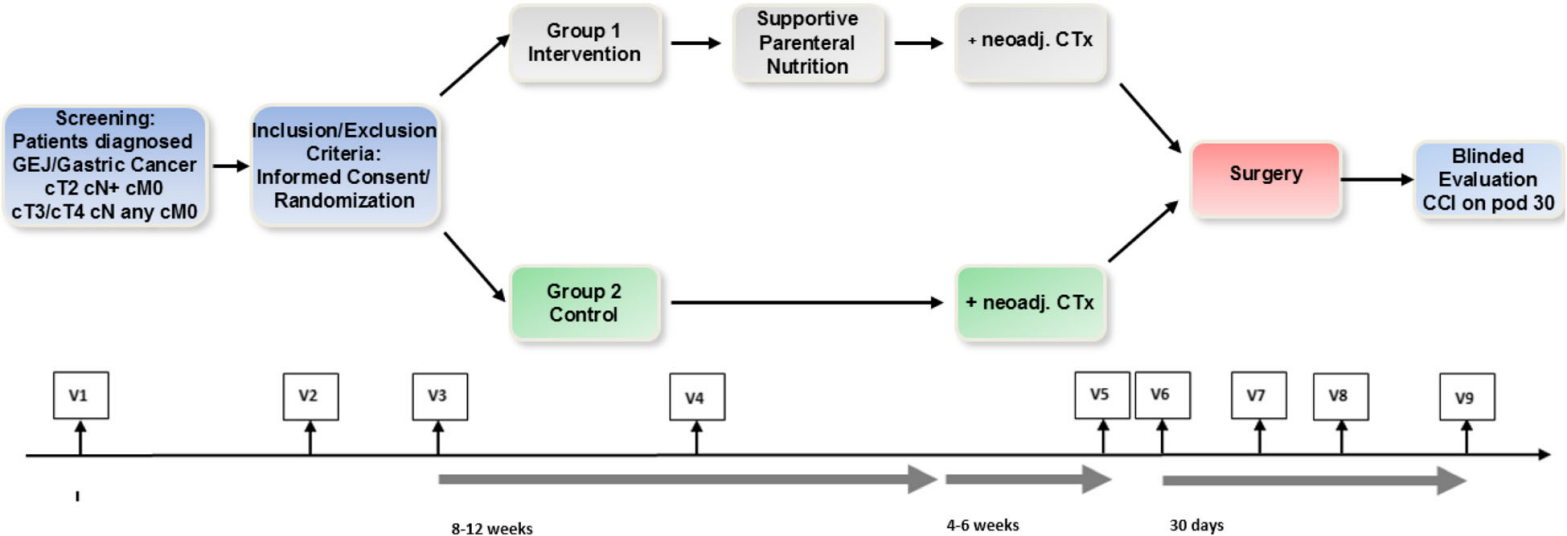
*Flowchart* of the PERCOG trial. CCI Comprehensive Complication Index, neoadj. neoadjuvant, Ctx chemotherapy, GEJ gastro-esophageal junction

**Fig. 2:**
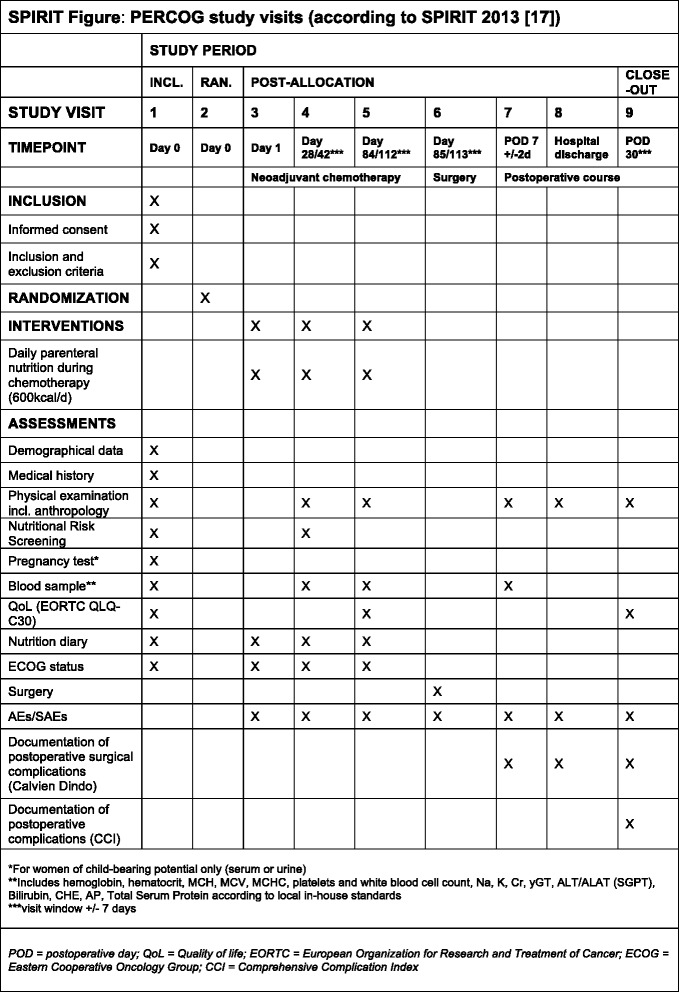
SPIRIT figure for the PERCOG trial

Only specialized oncological surgical centers with the required infrastructure will be included in the trial to achieve the target sample size. The recruitment period is set to 27 months (first patient in to last patient out 32 months). Figure 1 shows the trial flow scheme and Fig. 2 (SPIRIT figure) the schedule of enrolment, interventions, and assessments according to the SPIRIT 2013 statement [17]. The SPIRIT checklist for the study protocol can be found in Additional file 1.

### Randomization and blinding

Before inclusion in the trial, a GCP-certified investigator will perform the screening and detailed information of patients and will obtain their written informed consent. A member of the study team will perform randomization after completion of study visit 1. An online tool of the Munich Center of Clinical Trials (*Münchner Studien Zentrum* [MSZ]) will be used to generate the allocation sequence. This online tool uses pre-defined randomization lists, which will be created block-wise at the Institute of Medical Statistics and Epidemiology of the TUM. Basic characteristics of the patient and day of randomization must be documented on the printed randomization sheets. Subsequently, randomization sheets must be stored away from the patient records and trial documents to assure blinding. To ensure observer blinding, the assessment of primary and secondary outcomes will be performed by medical doctors, which are not otherwise integrated in the conduct of the trial and blinded to the group allocation of participants. Observers will be surgeons of spatially separated participating study centers, specifically trained in the assessment of the CCI. The observer training has to be documented on a separate training log. The observers will receive the patient’s surgical file only. Before sending the file, it has to be verified that this file contains data on the postoperative treatment only and no data that allow conclusions about the pre-surgical supportive treatment. All patient-related data and data concerning the study intervention have to be blanked out. The oncological file, in which the study specific treatment is documented, will not be available to the independent observers. To ensure blinding, the observers will not perform patient visits but assess the CCI on basis of the surgical file only. Blinding of patients is not feasible. Unblinding of the local principal investigator is permissible if significant hazards for individuals’ safety or welfare occur.

### Interventions

#### Experimental intervention

In the experimental group, patients will receive supportive parenteral nutrition containing 600 kcal per day via a central line (e.g. intravascular port system) in addition to their regular enteral nutrition. The enteral nutrition will be documented in a specific “nutrition diary.” For the parenteral nutrition, multi-chamber-bags containing amino acids, glucose, and lipids have to be used. On demand, vitamins, electrolytes, and micronutrients (trace elements) can be added. The choice of product is left to the local home care service and is not bound to a specific manufacturer. If the patient is unable to achieve his daily calorie requirement by enteral nutrition, the missing calories will be added to the 600 kcal of parenteral nutrition. A dietary assistant or a member of the study team will recalculate the daily calorie requirement once weekly, in the framework of the routine clinical evaluation during the NACT. The daily calorie requirement is calculated as follows:

- women: 25 kcal/kg of the ideal body weight (IBW)
- men: 30 kcal/kg of the IBW

The daily supportive parenteral nutrition will start with the first day of preoperative chemotherapy until the day of surgery. The administration will be effectuated by qualified nurses and can take place at the patient’s home if an ambulatory home care service is organized. The beginning of the supportive nutritional therapy will be documented in the electronic case report form (eCRF). In addition, the home care service will record the administration on a separate log.

#### Control intervention

The control group will not receive any supportive parenteral nutrition during the preoperative chemotherapy. The enteral nutrition will be documented in a specific “nutrition diary,” since it cannot be ruled out that the control group patients will increase their calorie intake due to the participation in this trial. To ensure that patients in the control group are not at risk for malnutrition during the chemotherapy, the NRS 2002 screening will be repeated at study visit 4. If patients are not able to cover > 25–50% of their daily calorie requirement by enteral nutrition alone, they will be provided with additional supportive parenteral nutrition as well.

### Permitted and not permitted medication(s)/treatment(s)

No additional study-specific treatments will be performed within the trial. If indicated for medical reasons, all kinds of medication are permitted during the trial. Postoperative medication with adverse effects on the immune system (e.g. corticoids and other immunosuppressive agents) will be recorded in the eCRF.

### Risks

Except for the administration of the parenteral nutrition in the experimental group and the survey of questionnaires, no additional study-specific examinations or interventions outside of the clinical routine will be applied. No additional risks for study patients are anticipated, since parenteral nutrition represents a clinically established standard method and all products are approved for this indication in Germany (in-label). Parenteral nutrition during chemotherapy of malnourished patients is explicitly recommended by current clinical guidelines [1]. The implantation of a central venous port system is required for the application of the preoperative chemotherapy. The blood samples will be taken during clinical routine evaluation of chemotherapy toxicity. The surgical procedure will be performed according to local standards. Adverse effects of parenteral nutrition include a slightly increased risk of port system infections. Therefore, only qualified personnel will perform the administration of parenteral nutrition in the PERCOG trial. This mildly increased risk is outweighed by the potential benefit of reduced postoperative complications by supportive parenteral nutrition, which has already been demonstrated in intensive care unit (ICU) patients [18]. The individuals’ safety is ensured by regular study visits, enforcing GCP guidelines. Furthermore, according to AMG, each patient will be provided with insurance coverage for any potential harms from trial participation. The study will be planned, conducted, and analyzed according to all relevant national and international rules and regulations (AMG [15], ICH-GCP [16], Declaration of Helsinki [4]).

### Outcome measures

The primary efficacy endpoint of this trial is the absolute value of the CCI [5] on postoperative day 30, after the oncological resection. All deviations of the regular postoperative course have to be documented and classified according to the Clavien Dindo Classification (Table 1) [19]. Using these data, the CCI has to be calculated by an online tool (http://www.assessurgery.com/about_cci-calculator/) and documented on a source datasheet that will subsequently be entered in the eCRF.

**Table 1:** Clavien Dindo classification of surgical complications [19]

Grade	Definition
Grade I	Any deviation from the normal postoperative course without the need for pharmacological treatment or surgical, endoscopic, and radiological interventions
Grade II	Allowed therapeutic regimens are: drugs as antiemetics, antipyretics, analgesics, diuretics, electrolytes, and physiotherapy. This grade also includes wound infections opened at the bedside
Grade III	Requiring pharmacological treatment with drugs other than such allowed for grade I complications Blood transfusions and total parenteral nutrition are also included. Requiring surgical, endoscopic, or radiological intervention
Grade IIIa	Intervention not under general anesthesia
Grade IIIb	Intervention under general anesthesia
Grade IV	Life-threatening complication (including CNS complications) requiring IC/ICU management
Grade IVa	Single organ dysfunction (including dialysis)
Grade IVb	Multiorgan dysfunction
Grade V	Death of a patient
Suffix “d”	If the patient suffers from a complication at the time of discharge, the suffix “d” (for “disability”) is added to the respective grade of complication. This label indicates the need for a follow-up to fully evaluate the complication

*CNS* central nervous system, *IC* intermediate care, *ICU* intensive care unit

In addition, the following outcome measures have been defined as secondary endpoint measures: (1) rate of serious adverse events (SAEs) and adverse events (AEs) during NACT according to the Common Terminology Criteria for Adverse Events (CTCAE v4.03.); (2) rate of AEs/SAEs associated with the administration of parenteral nutrition according to CTCAE v4.03; (3) morbidity and mortality within 30 days postoperatively according to the Calvien Dindo classification (Table 1) [19]; (4) quality of life (QoL) according to the results of EORTC (European Organization for Research and Treatment of Cancer) QLQ-C30 questionnaire; (5) change of weight and body mass index (BMI); (6) length of postoperative hospital stay; and (7) length of postoperative ICU say.

### Data collection and management

The applicable local regulations of data privacy protection will be followed. Before inclusion, patients will be informed that any patient-related data and materials will be appropriately pseudonymized (pursuant to GCP regulations [16]) and that these data may be used for analysis and publication purposes. All trial data will be recorded in eCRFs. After inclusion, baseline data (demographical data, medical history, anthropometric data) will be documented accordingly. A physical exam will be performed and a routinely taken blood sample analyzed (see Fig. 2 for included parameters). The EORTC QLQ-C30 questionnaire for the assessment of QoL will be documented and “nutrition diaries” will be handed out and explained to all participants. In case of women with childbearing potential, a pregnancy test will also be performed. During NACT, three study visits will be performed where ECOG score, as well as AEs and SAEs will be assessed and documented in the eCRF. At visit 3 (pre-chemotherapy), data on the type, duration, and dosage of chemotherapy and any additional radiotherapy will be documented. In addition, parameters of the planned parenteral nutrition intervention, the planned application route (e.g. via intravenous port system) and the used product will be documented for patients in the experimental group.

Furthermore, the IBW will be calculated as follows:

- IBW men: 50 kg + (2.3 kg * [body height (in cm) – 152.4 cm]/2.54 cm); and
- IBW women: 45.5 kg + (2.3 kg * [body height (in cm) – 152.4 cm]/2,54 cm).

Subsequently the required daily calorie supply can be calculated as:

- IBW * 30 kcal for men; and
- IBW * 25 kcal for women.

At visit 4 after the first half of chemotherapy, ECOG score, AEs, and SAEs will be assessed for all patients. In addition, anthropometric data will be reassessed and nutrition diaries will be collected and the enteral calorie intake documented. In the control group, the nutritional risk will be reassessed using the NRS 2002 screening. If control group patients fail to meet 25–50% of their calorie demand by enteral nutrition alone, parenteral nutrition will be initiated according to the procedures in the experimental group.

At study visit 5, before the surgical procedure, the duration, dosage, and end-date of NACT will be documented. In addition, ECOG score, AEs/SAEs, anthropometric data, and nutrition diaries will be assessed. A physical exam will be performed and routine laboratory values checked for anomalies. Furthermore, the EORTC QLQ-C30 questionnaire will be re-assessed. Documented parameters of the surgical procedure (visit 6) include the type and duration of the surgical procedure performed, as well as any intraoperative complications. Postoperatively, the local study personnel will perform three study visits on which physical exams will be performed, routine laboratory values checked, and all postoperative complications documented. Visit 8 on the day of hospital discharge additionally includes documentation of the length of ICU stay and the pathology report of the tumor (TNM stage). On visit 9, the primary endpoint will be assessed by independent observers, according to the procedures explained earlier. All postoperative complications, their duration, and severity will be documented according to the Clavien Dindo classification and subsequently used for the calculation of the CCI. In addition, a physical exam will be performed and the EORTC QLQ-C30 questionnaire reassessed.

To promote complete follow-up, patients who will already have been discharged from the hospital by the time of the study visits will be compensated for any travel expenses necessary to ensure participant retention. If, however, the patient is unable to attend visit 9 due to postoperative treatment in a rehabilitation facility or for other medical reasons, a standardized protocol for evaluation and documentation will have to be filled out by the treating physician.

At the end of the clinical study, all study-relevant data must be archived for at least ten years. Patient identification lists and patient files will be separately retained in the respective study sites. The infrastructure and the personnel for the data management will be provided by the Data Management Center at the MSZ, a member of the German Network of Coordinating Centers for Clinical Trials (*Koordinierungszentren für Klinische Studien* [KKS]). The eCRFs are checked for completeness, plausibility, and correctness at entry. The investigator is responsible for the accuracy and verifiability of each entry. Validating programs as well as individual inspection of data through the MSZ will ensure completeness, validity, and plausibility. Each remaining question or missing response is documented through data clarification requests, to which the investigator is obliged to respond as quickly as possible.

### Access to data and dissemination of results

Only the sponsor, personnel authorized by the sponsor, and the trial statistician will have access to the final dataset. Limited access for local investigators will be regulated in the Clinical Trial Agreement. After completion of the clinical study, the coordinating principal investigator will prepare a multicenter manuscript of the study results for publication in a reputable scientific journal. Authorship eligibility will be regulated in the Clinical Trial Agreement. The publication of the principal results from any single-center experience within the trial is not allowed until the preparation and publication of the multicenter results.

### Monitoring

Monitoring will be performed by the MSZ, an institution experienced in the monitoring of multicenter, surgical RCTs, in order to guarantee high quality of the study conduct and data retrieval. Monitoring will be carried out in accordance with ICH-GCP guidelines [16]. Monitors will visit all participating centers on a regular basis, starting with an initiation visit at each site. Furthermore, close-out visits are planned for each center. A monitoring manual describing the scope of the monitoring activity in detail will be provided. The monitor is authorized to compare trial documents and original data in adherence to data protection rights. The investigator provides direct access of patient’s documents/original source data to the monitor at any time. The monitor will also have regular contact to all participating centers and the sponsor. Furthermore, as part of quality assurance according to GCP, the sponsor and the competent health authorities have the right to audit/inspect the trial sites and any other institutions involved in the trial.

### Safety evaluation and reporting of adverse events

All AEs/SAEs from the moment of randomization until the last study visit have to be assessed, documented, and reported by the local clinical investigator or designated sub-investigator. Postoperative complications and surgical complications do not have to be documented as AE/SAE, but will be documented according to the Clavien Dindo Classification (visits 7–9). During preoperative chemotherapy (visits 2–5), changes of laboratory parameters that are expected because of the underlying illness and that are graded < 3 according to CTCAE v4.03 do not have to be reported as AEs.

SAEs have to be reported within 24 h and will be classified by intensity, outcome, and causality. Exceptions are: (1) hospital treatment planned before inclusion in the study; (2) planned admission to the hospital for NACT; and (3) hospital admissions because of the underlying disease as long as they are not because of an aggravation of the disease.

All SAEs will be subject to a second assessment by a designated person who will be independent from the reporting investigator and the trial sponsor. According to AMG and GCP regulations, the ethics committee and the competent federal health authority will be informed of all suspected unexpected serious adverse reactions (SUSARs) and all SAEs resulting in death or being life-threatening. Safety reports resuming all AE/SAEs have to be compiled regularly by the principal investigator to reassess the risk–benefit analysis.

If a female participant gets pregnant during the trial, the investigator has to report the pregnancy to the sponsor within 24 h after becoming aware of it. It is recommended to follow-up on the pregnancy and outcome.

### Statistical methods

The statistical analysis will be conducted by a group-allocation-blinded statistician from the Institute for Medical Statistics and Epidemiology of the TUM, in line with the ICH-GCP guidelines [16]. For the statistical analysis, SAS software version 9.2 or higher will be used (SAS Institute Inc., Cary, NC, USA).

#### Sample size calculation

The sample size was calculated (nQuery Advisor software version 7.0, Statistical Solutions Ltd., Cork, Ireland) based on the primary endpoints of the study. Assuming that the nutrition intervention can reduce the CCI by 10 points (with a standard deviation of 20), 69 patients have to be recruited in each group to achieve a power of 80% with a two-sided significance level of 5% using the Wilcoxon signed-rank test. Expecting a drop-out rate of 8% (based on experience from previous similar trials) a total of 150 (75 per group) patients have to be included in the study.

#### Analysis population

The primary and secondary endpoints will be analyzed on the intention-to-treat (ITT) set, consisting of all patients randomized in the study independent of the intervention they receive (analysis “as randomized”). The safety analysis will be performed on the safety set, consisting of all patients randomized into the study and assigned to the treatment group of their actual treatment.

#### Analysis of the primary endpoint measure

Preoperative supportive parenteral nutrition will be tested for superiority over no supportive nutrition during NACT with respect to the absolute value of CCI on postoperative day 30 using Fisher’s exact test with the following hypothesis:

$$ H0:a=0\  vs. HA:a\ne 0 $$

Where “a” denotes the difference of the distribution of CCI values in both patient groups. The distribution will be compared using the Wilcoxon signed-rank test. The test will be performed two-sided and with a global significance level of 5%.

#### Sensitivity of the primary endpoint measure

Missing primary endpoint data will be imputed conservatively. For sensitivity analysis, the primary endpoint analysis will be repeated in the per-protocol (PP) population. The PP population includes all patients of the ITT population “as treated.” Patients who did not complete visit 9 will be excluded.

#### Analysis of secondary endpoint measures

Secondary endpoints will be analyzed by treatment arm on the ITT set, using appropriate descriptive statistics. Any explorative statistical testing will be performed using a significance level of 5%.

#### Safety analysis

All AE/SAEs will be analyzed with incidence rates by treatment group and according to their severity. All AE/SAEs rated as related to the study treatment will be listed separately. For the comparisons between groups, the Chi-square test and Fisher’s exact test will be used if appropriate. All patients who started visit 2 will be analyzed “as treated.”

### Withdrawals

Patients are free to withdraw from trial participation at their own request at any time and without giving reasons for their decisions. Withdrawals will be documented in the eCRF and in the patient’s medical record. However, all ongoing SAEs must be followed up and documented until their final outcome can be determined. Patients that withdraw their consent or are excluded from the trial due to the decision of the sponsor or investigator will be replaced to ensure a sufficient sample size. Drop-outs (e.g. lost to follow up) have been taken into account at sample size calculation and will not be replaced.

### Stopping guidelines

The trial can be prematurely closed by the sponsor in consultation with the responsible biostatistician for the following reasons: (1) it appears that patients’ enrolment is unsatisfactory with respect to quality or quantity, or data recording is severely inaccurate or incomplete; and (2) there is external evidence demanding a termination of the trial, e.g. indicating that the rate or severity of SAEs or morbidity in this trial poses a potential health hazard caused by the intervention in one of the trial groups. In case of premature closure, the ethics committee and health authorities must be informed.

### Registration

The trial protocol was registered at the German Clinical Trials Register (part of the WHO International Clinical Trials Registry Platform) under number DRKS00009451 on 3 July 2017.

### Good clinical practice

The procedures set out in this trial protocol, pertaining to the conduct, evaluation, and documentation of this trial, are designed to ensure that all persons involved in the trial abide by GCP [16] and the ethical principles described in the current version of the Declaration of Helsinki [4]. The trial will be carried out in compliance with German Drug Law (AMG) [15] as well as all other local legal and regulatory requirements.

## Discussion

The PERCOG trial has a pragmatic, two-armed study design (parenteral nutrition vs no parenteral nutrition during NACT) and shall be conducted in four centers within the German Surgical Trial network CHIR-*Net*. A multicenter approach was chosen to increase external validity. Internal validity and data quality assurance are established by adherence to the SPIRIT guideline and GCP regulations, regarding recruitment, training of study personnel, methods against bias, outcome reporting, and documentation.

All patients suffering from non-metastatic gastric or esophago-gastric cancer, staged at least uT2N+ receiving NACT, will be screened within 27 months. Specialized oncological surgical centers were chosen and broad inclusion criteria were applied to ensure rapid and sufficient recruitment to achieve the target sample size.

The primary endpoint is the postoperative CCI, which was recently demonstrated to be a superior and sensitive outcome measure for RCTs in surgery [5]. Since reporting of postoperative complications is often non-standardized and incomplete, surgical trials can be difficult to compare. The CCI includes all types of postoperative complications independent of their severity and has been shown to be more sensitive than other existing morbidity endpoints [3–5].

The results of this pragmatic trial will provide high-level evidence for clinical recommendations regarding the effectiveness of preoperative parenteral nutrition during NACT of patients with gastric or esophago-gastric cancer.

### Trial status

Recruitment is planned to start in January 2018.

## Additional file

Additional file 1: SPIRIT 2013 checklist. (PDF 104 kb)

## Abbreviations

AE: Adverse event
AMG: *Arzneimittelgesetz*/German Drug Law
ASA: American Society of Anesthesiologists
BMI: Body mass index
CCI: Comprehensive Complication Index
COPD: Chronic obstructive pulmonary disease
CTCAE: Common Terminology Criteria for Adverse Events
ECOG: Eastern Cooperative Oncology Group
eCRF: Electronic case report form
EORTC: European Organization for Research and Treatment of Cancer
GCP: Good Clinical Practice
GIT: Gastrointestinal tract
ICH: International Conference on Harmonization of Technical Requirements for Registration of Pharmaceuticals for Human Use
ICU: Intensive care unit
ITT: Intention-to-treat
KKS: *Koordinierungszentren für Klinische Studien*/German Network of Coordinating Centers for Clinical Trials
MSZ: *Münchner Studienzentrum*/Munich Centre of Clinical Studies
NRS: Nutritional Risk Score
NYHA: New York Heart Association
PP: Per protocol
QoL: Quality of life
RCT: Randomized controlled trial
SAE: Serious adverse event
SUSAR: Suspected unexpected serious adverse reaction
TUM: *Technische Universität München*/Technical University of Munich
